# Editorial: Mechanistic, machine learning and hybrid models of the ‘other’ endocrine regulatory systems in health and disease, volume II

**DOI:** 10.3389/fendo.2026.1806508

**Published:** 2026-03-03

**Authors:** Joseph DiStefano, Johannes W. Dietrich, Uri Alon

**Affiliations:** 1 Departments of Computer Science and Medicine, University of California at Los Angeles, Los Angeles, CA, United States; 2 Diabetes, Endocrinology and Metabolism Section, Department of Internal Medicine I, St. Josef University Hospital, Ruhr University Bochum, Bochum, Germany; 3 Diabetes Centre Bochum/Hattingen, Klinik Blankenstein, Hattingen, Germany; 4 Centre for Rare Endocrine Diseases, Ruhr Centre for Rare Diseases (CeSER), Ruhr University Bochum and Witten/Herdecke University, Bochum, Germany; 5 Centre for Diabetes Technology, Catholic Hospitals Bochum, Ruhr University Bochum, Bochum, Germany; 6 Department of Molecular Cell Biology, Weizmann Institute of Science, Rehovot, Israel

**Keywords:** computer simulation, hybrid mechanistic-machine learning model, machine learning model, mathematical modelling, mechanistic model, stress hyperglycemia, thyroid, uric acid

**Figure 1:**
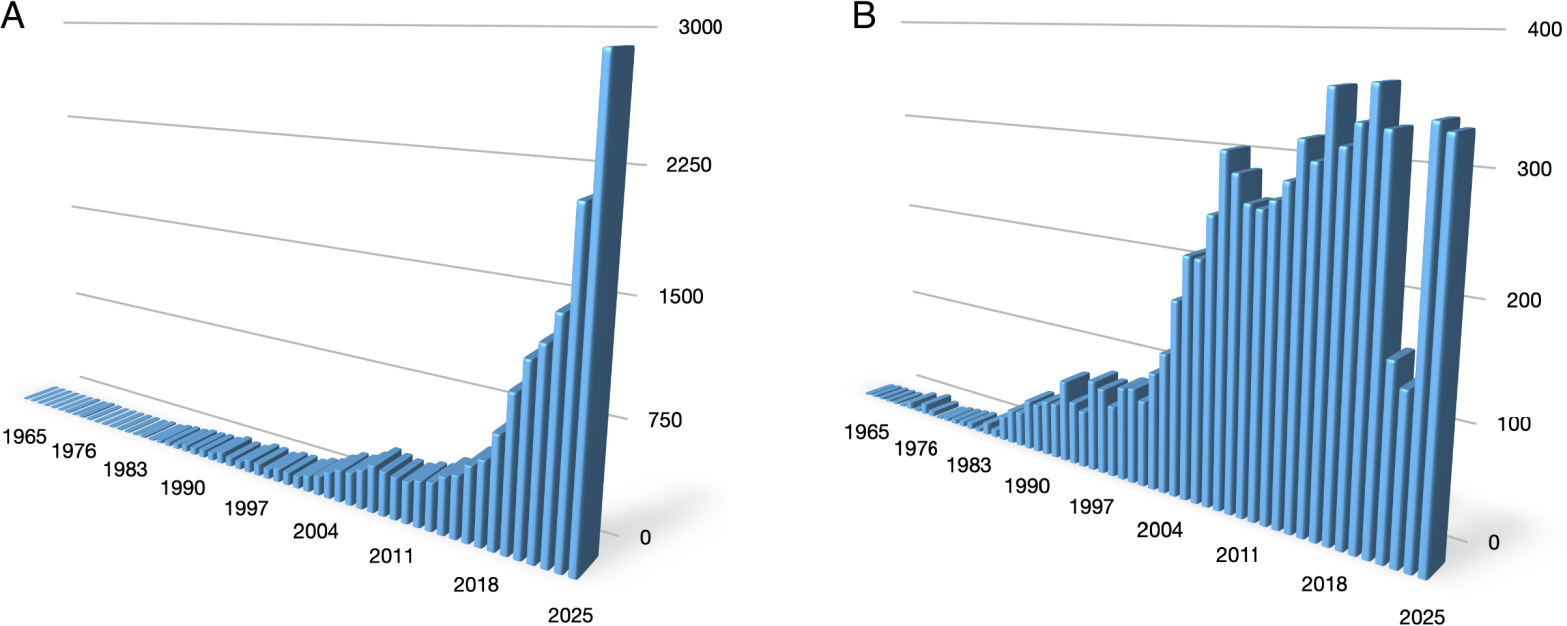
Yearly numbers of publications in PubMed on mathematical modelling in endocrinology and diabetes with **(A)** and without **(B)** machine learning. Search terms were “(“mathematical modelling” OR “computer simulation” OR “machine learning”) AND (endocrinology OR endocrine OR hormone OR hormonal OR diabetes)” for **(A)** and “(“mathematical modelling” OR “computer simulation”) AND (endocrinology OR endocrine OR hormone OR hormonal OR diabetes)” for **(B)**.

Mathematical modelling, computer simulations, and machine learning are receiving growing attention in the scientific literature of endocrinology (**Figure 1A**). Most of the recent, nearly exponential growth is due to publications on machine learning (ML), but even without ML, the increase was substantial after the year 2000 (**Figure 1B**).

A large portion of the mentioned publications deals with insulin-glucose homeostasis and diabetes mellitus. Mathematical modelling is an established technique in today’s diabetology, and the models deliver reliable methods for research (1, 2), differential diagnosis (3–6) and diabetes technology (7–9).

Other subfields of endocrinology have also benefited from modelling (10–12). More progress is to be expected, the more so as recent developments in theoretical foundations have promoted advances in the respective applications (13). Compared to diabetology, this occurred, however, on a much smaller scale (14).

These alternative implementations deserve more attention. This is the reason why we initiated this second volume of the Research Topic on the “other” endocrine regulatory systems. It received submissions that span a relatively wide field, ranging from urate metabolism to stress-induced metabolic alterations and neuroendocrine interactions, as well as thyroid homeostasis.

Hyperuricaemia is a common metabolic disease that may lead to significant complications, including renal failure and gouty arthritis. It is strongly associated with cardiometabolic syndrome (syndrome X), and it is assumed that elevated uric acid concentration is both a cause and consequence of the metabolic syndrome and its components (obesity, dyslipidaemia, hypertension and impaired glucose homeostasis) (15–17). In this Research Topic, Ma et al. present the results of a scientometric analysis that confirms this relationship with respect to diabetes mellitus. It uncovers research trends regarding possible molecular mechanisms and potential future treatment strategies.

The transition from asymptomatic hyperuricaemia to gout is ignited by several positive feedback loops, linking inflammation to the production and excretion of uric acid (18–21). Resveratrol has been shown to be beneficial in hyperuricaemia and gout, but the mechanisms underlying its action remain unknown. By combining modelling techniques of network pharmacology with metabolomics, Xu et al. demonstrated that resveratrol has anti-inflammatory actions mediated via the NF-κB, MAPK and JAK/STAT3 signalling pathways. This effect not only inhibits the acute inflammatory response in gout attacks but also reduces the concentration of uric acid. The disruption of pathological positive feedback loops could result in favourable short- and long-term effects.

Sympathetic tonus and nearly all stress hormones, e.g. catecholamines, glucocorticoids, growth hormone and (as mediators for type 2 allostatic load, certain thyroid hormones), raise the blood glucose concentration via induction of insulin resistance. Likewise, activation of a positive feedback loop linking proinflammatory cytokines and glucose concentration causes hyperglycaemia in inflammation (22–25). In critical illness, the stress hyperglycaemia ratio (SHR), calculated by dividing the admission glucose concentration by the average glucose concentration (estimated from the HbA1c fraction), helps to distinguish the effects of acute stress from chronic hyperglycaemia (26). The value of the SHR is, however, understudied in sepsis. The study by Xia et al. evaluated the effect of SHR on fatality in more than 2400 patients affected by sepsis with the Boruta algorithm and methods of survival analysis. They found a complex and nearly U-shaped relationship between SHR and fatality, with the most favourable prognosis around a ratio of 0.9.

Hormones of different classes profoundly affect the function of the central nervous system, and, conversely, the endocrine system is controlled by the brain (27–30). Specifics of this bidirectional relationship are, in many cases, poorly understood, however. Even where more details have been elucidated, it is unknown how to leverage this knowledge for clinical decision-making and therapeutic interventions. The paper by Liu suggests the Hormone Interaction Dynamics Network (HIDN), a framework for modelling and analysing the interaction between endocrine feedback control systems and EEG signals. It combines differential equations, graph-based neural network methods, machine learning and encoding of external stimuli to model the control of hormone secretion and inter-gland interactions. On this basis, a second framework, the Adaptive Hormonal Regulation Strategy (AHRS), is proposed. It aims at the development of advanced intervention techniques. In validation studies with existing datasets, the author demonstrates that the new framework predicts the interaction of EEG signals with allostatic hormonal dynamics after emotional stimuli is better than state-of-the-art methods.

On the basis of a long continuity of thyroid modelling, DiStefano et al. present an extended version of the app THYROSIM, a simulator of the hypothalamus-pituitary-thyroid axis. Its foundations are in a succession of models for thyroid homeostasis that spans nearly six decades (31, 32). In 2022, the group extended THYROSIM to p-THYROSIM, thereby providing options for personalisation (33). In the updated version of p-THYROSIM included in this series, earlier assumptions about plasma volume sex differences and T3 degradation were corrected, and the new model was developed into an app that runs on the iPhone and iPad, as well as a 1000-day Python program version for longer-term disease progression. Simulations with p-THYROSIM confirm the observation that for a subgroup of patients with hypothyroidism, a combination therapy of levothyroxine (L-T4) and liothyronine (L-T3) may be more effective than monotherapy with L-T4. This insight may be important for the large group of patients with persisting symptoms and reduced quality of life despite normal TSH concentration under L-T4, an increasingly recognised condition referred to as syndrome T or SORSHOT (34, 35).

The papers summarised in this Research Topic cover a broad range of topics that include diverse areas like glucose homeostasis in stress, hyperuricaemia and gout, interactions between the central nervous system and hormonal feedback loops, and thyroid homeostasis. Likewise, the methods used are multifaceted, including scientometrics, network pharmacology, animal experiments, metabolomics, feature selection with random forests, neural networks, machine learning, systems modelling, compartment analysis and computer simulations. With this broad foundation, they provide an overview of the state of the art and potential perspectives for directions of future research.
